# An evaluation of perceptions and implementation of good review practices in the medicine review process at the Egyptian drug authority

**DOI:** 10.3389/fmed.2026.1920714

**Published:** 2026-10-08

**Authors:** Ghada Zaki, Sam Salek, Stuart Walker, Sungsoo Chun

**Affiliations:** 1 Department of Biological, Chemical, and Global Health Science, School of Science and Engineering, the American University in Cairo, New Cairo, Egypt; 2 School of Health, Medicine and Life Sciences, University of Hertfordshire, Hatfield, United Kingdom; 3 Centre of Innovative Regulatory Science, London, United Kingdom

**Keywords:** Egyptian Drug Authority (EDA), gap analysis, Good Review Practice (GRevP), maturity level 4, regulatory review, strengthning regulatory system

## Abstract

**Introduction:**

Good Review Practices (GRevPs) are considered of value for regulatory system strengthening that support timely, consistent, transparent, and high-quality regulatory reviews. It is therefore important that GRevPs are continuously updated and monitored to improve the review process. This study is the first assessment of GRevPs within the Egyptian Drug Authority (EDA) and provides a baseline for future monitoring and improvement.

**Methods:**

A cross-sectional study was conducted among Marketing Authorisation (MA) assessors within the EDA using a semi-structured questionnaire. It comprised two sections: the validated Good Review Practices (GRevPs) questionnaire developed by the Centre for Innovation in Regulatory Science (CIRS), which assessed knowledge, attitudes, practices and barriers to GRevPs adoption; and a study-specific section assessing the perceived importance and implementation of review-quality activities. Descriptive statistics summarised the data, while a gap analysis using mean differences and an Importance-Performance Analysis (IPA) matrix identified priority areas for improvement. Open-ended responses were analysed using conventional content analysis.

**Results:**

Fifty-five of the 80 assessors approached completed the questionnaire (69%). Within the MA departments, respondents reported comparable levels of full and partial adoption of GRevPs (44% and 53%, respectively). Assessors demonstrated a good understanding of GRevPs and identified efficiency, consistency, reduced duplication, and error minimisation as key benefits. Gap analysis showed that review-quality activities were recognised as important, with positive practices and attitudes toward GRevPs. However, areas requiring further improvement included continuous, practice-oriented training; proactive communication with applicants; adherence to timelines; cross-team coordination; consistent recording and updating of review information; and mechanisms for acting on applicant feedback. High workload, limited staffing capacity, time pressure, and communication challenges were identified as the main barriers to implementation.

**Discussion:**

These findings indicate that EDA has established a strong foundation for implementing GRevPs, supported by documented procedures, review tools, and assessors’ understanding. However, greater attention is needed to ensure consistent implementation, monitoring, and continuous improvement of GRevPs, particularly through ongoing practical training, digitalisation, external tracking mechanisms, and adequate resource allocation. As the Egyptian Drug Authority (EDA) progresses towards Maturity Level 4 and WHO-Listed Authority aspirations, understanding how GRevPs are perceived and implemented in practice becomes increasingly important.

## Introduction

As noted by the World Health Organisation (WHO), an effective medicine regulatory system is a fundamental building block of a resilient and sustainable healthcare system and supports improved public health outcomes (1). As such, it is both important and urgent to establish robust regulatory systems that enable faster access to and a continuous supply of safe, high-quality medicines, while ensuring rigorous scientific standards of review and oversight are maintained throughout the product's lifecycle (2, 3). In the current globalised environment, National medicine regulatory authorities (NRAs) are actively working to build regulatory systems that are patient-centric, fit for purpose, adaptable to national contexts, responsive to emerging technologies, new product types, and evolving patient needs (4–6) At the same time, they must manage increasing workloads with limited resources by making the best use of available expertise and focusing regulatory efforts where they are most needed (6). In response, authorities are seeking to streamline and optimise regulatory processes, raise quality standards, and adopt more efficient and sustainable practices to advance performance and enable continuous improvement (2, 4, 6).

One such regulatory process is good review practices (GRevPs), defined by the World Health Organisation (WHO) as follows: “GRevPs are documented best practices for any aspect related to the process, format, content, and management of a medical product review” (7). They represent a set of enabling elements, practices, and activities that guide how regulatory reviews are organised, managed, standardised, and documented to build and improve the quality of review processes, reports, and decision outcomes. The main objective of GRevPs is to enable high-quality regulatory reviews, as reflected not only in timely review and approval processes but also in key quality attributes such as transparency, predictability, consistency, and quality decisions (7, 8).

In practice, GRevPs are an integrated system of tools, processes, organisational enablers and behaviors designed to achieve quality regulatory reviews. They are implemented through formalised and documented procedures, such as standard operating procedures, guidelines, and assessment templates, supported by learning activities including training and mentoring, as well as monitoring measures, such as audits, to ensure consistent application (7). At the organisational level, quality measures, including a quality policy and a dedicated quality unit, to enable the building and consistent application of these practices across agencies (9). Finally, GRevPs also reflect a philosophy and culture of quality, and their effectiveness depends on how well assessors understand, accept, and integrate them into their daily work. According to the Centre of Innovation and Regulatory Science (CIRS), “It is important that staff understand the concepts of GRevPs and put these into action rather than just acknowledge their existence" (4).

The importance of practice of GRevPs is recognised globally. The WHO encourages regulatory agencies to integrate them into their systems to strengthen regulatory performance. Further, their inclusion as a key sub-indicator (MA04.10: regulations and guidelines for GRevPs are developed, recognised and implemented within the WHO Global Benchmarking tool highlights their role in assessing the maturity and performance of regulatory review functions (10). By embedding quality systematically into review processes, authorities can strengthen confidence in the quality of their assessments and assessment reports, thereby supporting collaboration, reliance, and work-sharing across regulatory systems (7).

Egypt has the largest pharmaceutical market in Africa, accounting for more than 27% of the continent's pharmaceutical sector (11). It also ranks second in the Middle East and Africa (MEA) region, after the Kingdom of Saudi Arabia (KSA), with over 12,000 registered medicinal products currently on the market (11). The Egyptian Drug Authority (EDA) established in 2019 as an independent governmental body responsible for regulating medical products in Egypt, has significantly strengthened its regulatory system in recent years (12). The EDA achieved WHO Maturity Level 3 (ML3) status for both medicines and vaccines regulation, making Egypt the first country in Africa to attain ML3 for both areas. As of August 2026, Egypt is one of ten African countries whose national medicines regulatory authorities (NMRAs) have achieved ML3 status. These include regulatory authorities in Ghana, Ethiopia, Mozambique, Nigeria, Rwanda, Senegal, South Africa, Tanzania, and Zimbabwe (13). Furthermore, EDA is actively engaged with international regulatory agencies and organisations to align with global standards and exchange information on best regulatory practices. As the EDA continues to strengthen its regulatory performance and progress toward higher levels of maturity, including Maturity Level 4 and WHO-Listed Authority (WLA), it becomes increasingly important to evaluate regulatory processes and practices to identify areas for improvement and support advancement toward higher regulatory maturity*.* The aim of this study, therefore, was to evaluate the implementation and practice of Good Review Practices within the EDA from the perspective of its medical assessors.

The specific objectives of this study were to: establish a baseline of assessors’ perceived knowledge, attitudes, and practices towards GRevPs; explore assessors’ current perceptions of the adoption level and benefits of GRevPs; examine the operational tools, procedures, and the learning and monitoring mechanisms currently in place to support GRevPs implementation; assess the perceived importance and level of implementation across key domains of quality review; and identify perceived barriers to GRevPs implementation and areas for improvement.

## Methods

### Research design and assessment tool

This study used a cross-sectional design and was conducted at the Egyptian Drug Authority (EDA) between October 2025 and February 2026. Data were collected using a semi-structured questionnaire with two sections: the validated Good Review Practices (GRevPs) questionnaire developed by the Centre for Innovation in Regulatory Science (CIRS) and an additional study-specific section.

**Section 1**: The validated GRevPs questionnaire has previously been used to characterise the development and embeddedness of GRevPs across medicine regulatory authorities (4, 14, 15). The tool includes mandatory structured questions assessing regulatory assessors’ knowledge, attitudes, and practices relating to GRevPs and an optional free-text section for additional comments.

The original GRevPs questionnaire was reviewed and pilot- tested with three senior assessors within the EDA to evaluate its clarity, readability, and suitability for use. Based on the feedback received, a guidance note was produced to provide clarifications through explanatory text to improve the understanding of selected questions.

**Section 2:** The study-specific additional section was developed to complement the original questionnaire and provide operational insights into the perceived importance and implementation of selected review–quality activities. It included 31 activities grouped into six domains: quality management (7 items), transparency (4 items), review conduct (6 items), consistency (4 items), communication (6 items), and stakeholder engagement (4 items). The activities and domains were identified with reference to WHO guidance on Good Regulatory Practices (6) and Good Review Practices (7), as well as the CIRS quality scorecards (16) and the R&D Briefing 58 on GRevPs (8).

Each activity was assessed using two parallel five-point Likert scales: one measuring perceived importance (1 = least important to 5 = most important) and the other measuring perceived level of implementation (1 = least implemented to 5 = highly implemented). The content validity of the additional section was reviewed for clarity and suitability and assessed using the Item-Level Content Validity Index (I-CVI) and the Scale-Level Content Validity Index (S-CVI) (17). Three senior EDA assessors independently rated the relevance of each item on a four-point scale (1 = not relevant, 2 = somewhat relevant, 3 = quite relevant, and 4 = highly relevant). Internal consistency was further assessed using Cronbach's *α*.

### Participants and sampling

The study population consisted of approximately 220 assessors working within the Marketing Authorisation (MA) departments for human pharmaceuticals and biological products at the Egyptian Drug Authority (EDA). The minimum required sample size was estimated using an online sample size calculator (18) based on Cochran's formula with finite population correction (19). Assuming a 95% confidence level, a 50% response distribution, and a 10% margin of error, the required sample size was 68 assessors. To allow for expected non-response and the limited accessibility of assessors within a specialised national regulatory authority, the target sample was increased to 80 assessors. Eligible participants were assessors working in the Marketing Authorisation (MA) departments for human pharmaceuticals and biological products, with at least six months’ experience to ensure sufficient exposure to Good Review Practices (GRevPs), and who provided informed consent to participate. Assessors from other regulatory functions, such as inspection, pharmacovigilance, and laboratory departments, were not eligible to participate.

Assessors meeting the eligibility criteria were approached directly through repeated visits to the MA departments, supplemented by electronic distribution of the questionnaire and follow-up reminders. Fifty-five assessors, of the 80 approached, completed the questionnaire, giving a response rate of 68.8%.

### Data analysis

#### Quantitative data analysis

Data were analysed using Microsoft Excel for data organisation, frequency and percentage calculations, and graphical presentation. R software (via R Commander) was used for statistical analyses.

Cross-tabulation and Fisher’s exact test were used to explore the association between respondents’ role level and years of experience (independent variables) and perceived GRevPs adoption at the authority and departmental levels (outcome variables).

For each of the 31 activities, mean importance and implementation values were calculated. Although Likert-scale responses are ordinal, mean values were used for descriptive and comparative analyses, consistent with published evidence supporting the use of means for five-point Likert-scale data (20–22). Paired t-tests were used to identify statistically significant differences between importance and implementation ratings (*p* < 0.05). Gap scores were calculated as the difference between the mean importance and implementation ratings. Previous studies across different research areas have similarly used mean rating differences to quantify identified gaps (23–25). Further, gap scores were categorised into three levels using quartile thresholds based on the distribution of the observed gap scores. Scores below Q1 indicated good alignment between perceived importance and implementation, scores from Q1 to < Q3 indicated moderate alignment requiring monitoring, and scores at or above Q3 indicated areas requiring prioritised improvement.

As a supplementary analysis, Importance–Performance Analysis (IPA) was used to visualise priority areas for improvement (26). Mean importance values were plotted on the vertical axis and mean implementation values on the horizontal axis. The overall mean importance and implementation scores across the activities were used to divide the matrix into four quadrants, with activity-level means above or below these values classified as high or low, respectively. Activities were classified into four quadrants: Quadrant I, Concentrate here (high importance, low implementation); Quadrant II, Keep up the good work (high importance, high implementation); Quadrant III, Low priority (low importance, low implementation); or Quadrant IV, Possible overkill (low importance, high implementation). Quadrant I was the primary focus for identifying priority areas for improvement.

#### Qualitative data analysis

Optional open-ended responses were analysed using conventional content analysis (27). Two researchers independently reviewed the responses and grouped similar comments into themes. Where applicable, illustrative quotations were reported with unique assessor identification numbers to preserve anonymity.

### Ethical consideration

The survey was approved by the Institutional Research Board at the American University in Cairo (CASE #2024-2025-285). All data were anonymised, no personal identifiers were collected, and respondents provided informed consent electronically before being enrolled in the study.

## Results

A total of 55 assessors from the Marketing Authorisation (MA) departments participated in the study, representing 69% (*n* = 80) of the assessors approached. All questions were mandatory, except for the open-ended comment section, resulting in a complete dataset with no missing responses. Participants represented different role levels and years of experience (Table 1).

**Table 1:** Demographic characteristics of the study participants.

Variable	Category	n	Percentage
Years of Experience	<2 years	4	7.27%
	2–5 years	14	25.45%
	>5–10 years	19	34.55%
	>10 years	18	32.73%
Role Level	Junior Assessor	17	30.91%
	Senior Assessor	18	32.73%
	Team Leader/ Manager	13	23.64%
	Head of Department	5	9.09%
	Director	2	3.64%

For the purpose of clarity, the results are presented in six parts: Part I—Knowledge, which describes Marketing Authorisation (MA) assessors’ perceptions of GRevPs adoption at two levels: across the wider EDA and within their own MA departments, as well as the perceived benefits of quality measures; Part II—Practice, which reports the operational tools and mechanisms supporting the implementation of GRevPs, their development, communication and monitoring to ensure their consistent use; Part III—Attitude, which covers assessors’ satisfaction with the framework and its implementation, and their views on how effectively these practices support their work; Part IV—Gap Analysis, comparing the perceived importance and implementation of review-quality activities; Part V—Perceived Barriers, identifying perceived barriers to effective GRevPs implementation; and Part VI—Qualitative Insights, presenting analysis of assessors’ free-text comments.

### Part I—knowledge

At the wider authority level, the majority of assessors perceived GRevPs as partially adopted (53%, *n* = 29), with a considerable proportion reporting full adoption (25%, *n* = 14), followed by limited adoption (11%) and insufficient knowledge (11%). At the departmental level, comparable proportions of respondents reported full adoption (44%, *n* = 24) and partial adoption (53%, *n* = 29) within their own departments, suggesting that GRevPs are widely integrated into the daily practice of MA departments.

Perceived GRevPs adoption was significantly associated with assessors’ role level and years of regulatory experience at both the authority and departmental levels (Table 2, *p* < 0.05). Among junior assessors, 11.8% (EDA) and 17.6% (department) reported full adoption, compared with 57.1% (EDA) and 85.7% (department) among directors and department heads. A similar pattern was observed across years of regulatory experience. Among assessors with less than two years of regulatory experience, 25.0% (EDA) and 0% (department) reported full adoption, compared with 50.0% and 83.3%, respectively, among those with more than ten years of experience (Table 2).

**Table 2:** Association between adoption level of GRevP and respondent characteristics.

Variable	Category	EDA–wide adoption (%)			MA Department Adoption (%)		
		Limited	Partial	Full	Limited	Partial	Full
Role Level	Junior Assessor	47.1	41.2	11.8	5.9	76.5	17.6
	Senior Assessor	5.6	72.2	22.2	5.6	50.0	44.4
	Team Leader/ Manager	15.4	53.8	30.8	0.0	46.2	53.8
	High management^a^	14.3	28.6	57.1	0.0	14.3	85.7
	*P*-value	0.035			0.04		
Years of Experience	<2 years	25	50	25	0	100	0
	2–5 years	50	42.9	7.1	7.1	85.7	7.1
	>5–10 years	15.8	68.4	15.8	5.3	52.6	42.1
	>10 years	5.6	44.4	50	0	16.7	83.3
	*P*-value	0.016			<0.001		

Limited adoption combines limited adoption and no knowledge to judge.

*P*-values were calculated using Fisher's exact test.

a High Management combines Head of Department and Director Categories due to small sample sizes.

The most frequently reported reasons for introducing quality measures were ensuring consistency in the process and decision-making (69%), improving process efficiency (47%), and reducing duplication or unnecessary steps (47%). Other commonly reported benefits included minimising errors (44%), increasing process predictability, and enhancing transparency (Figure 1).

**Figure 1:**
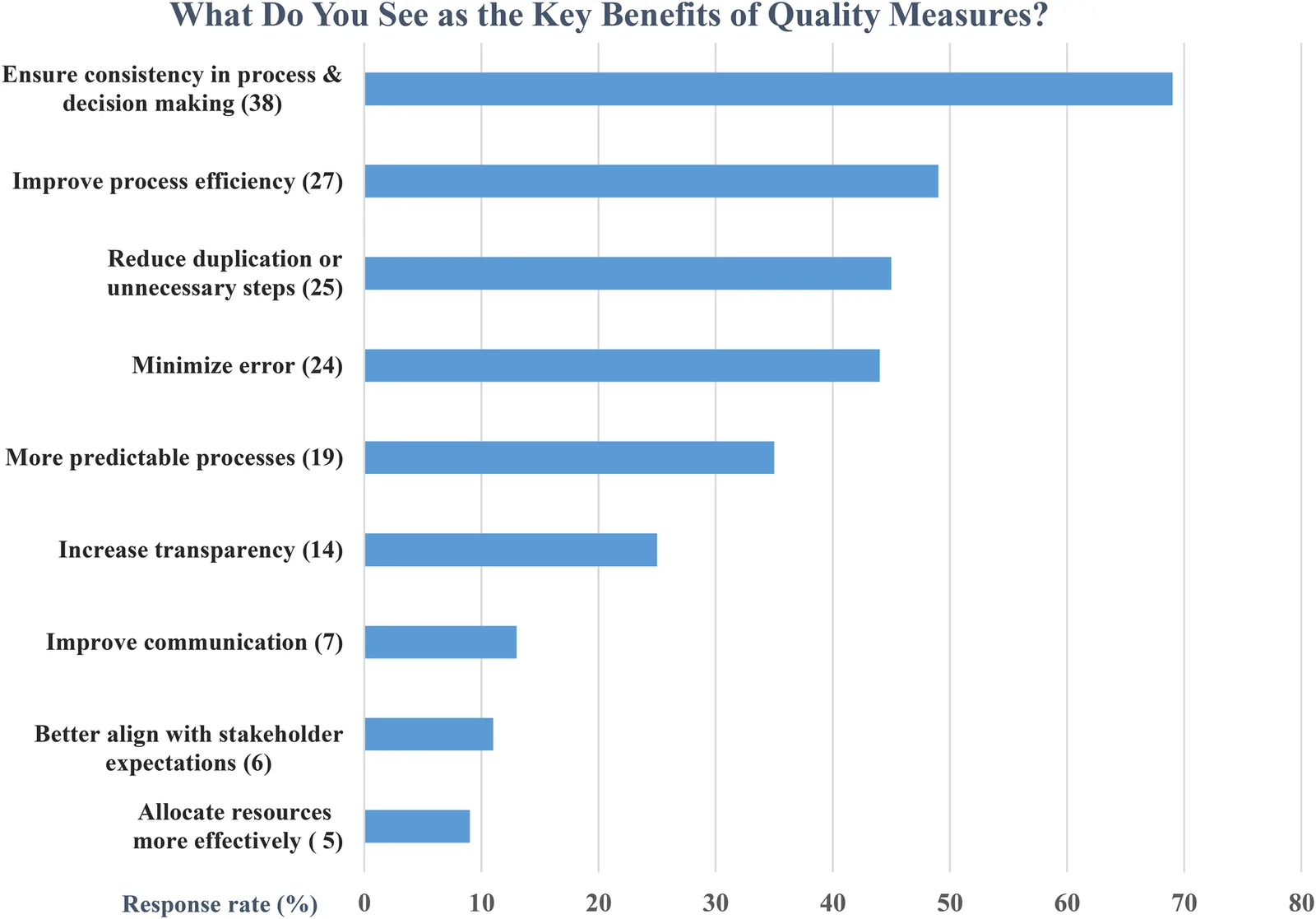
Perceived key benefits of implementing quality measures within EDA. Note: Percentages are based on 55 respondents; numbers in parentheses indicate the number of respondents selecting each option. Multiple selections were allowed.

### Part II—practices

The most commonly reported mechanisms supporting GRevPs implementation were the use of standard operating procedures to guide specific tasks (85%) and the distribution of general guidelines outlining review processes (60%). Structured training provided by colleagues and induction training for new staff were also selected as additional mechanisms supporting implementation. Formal training programmes, external sessions, and intranet-based learning were less frequently selected (Figure 2).

**Figure 2:**
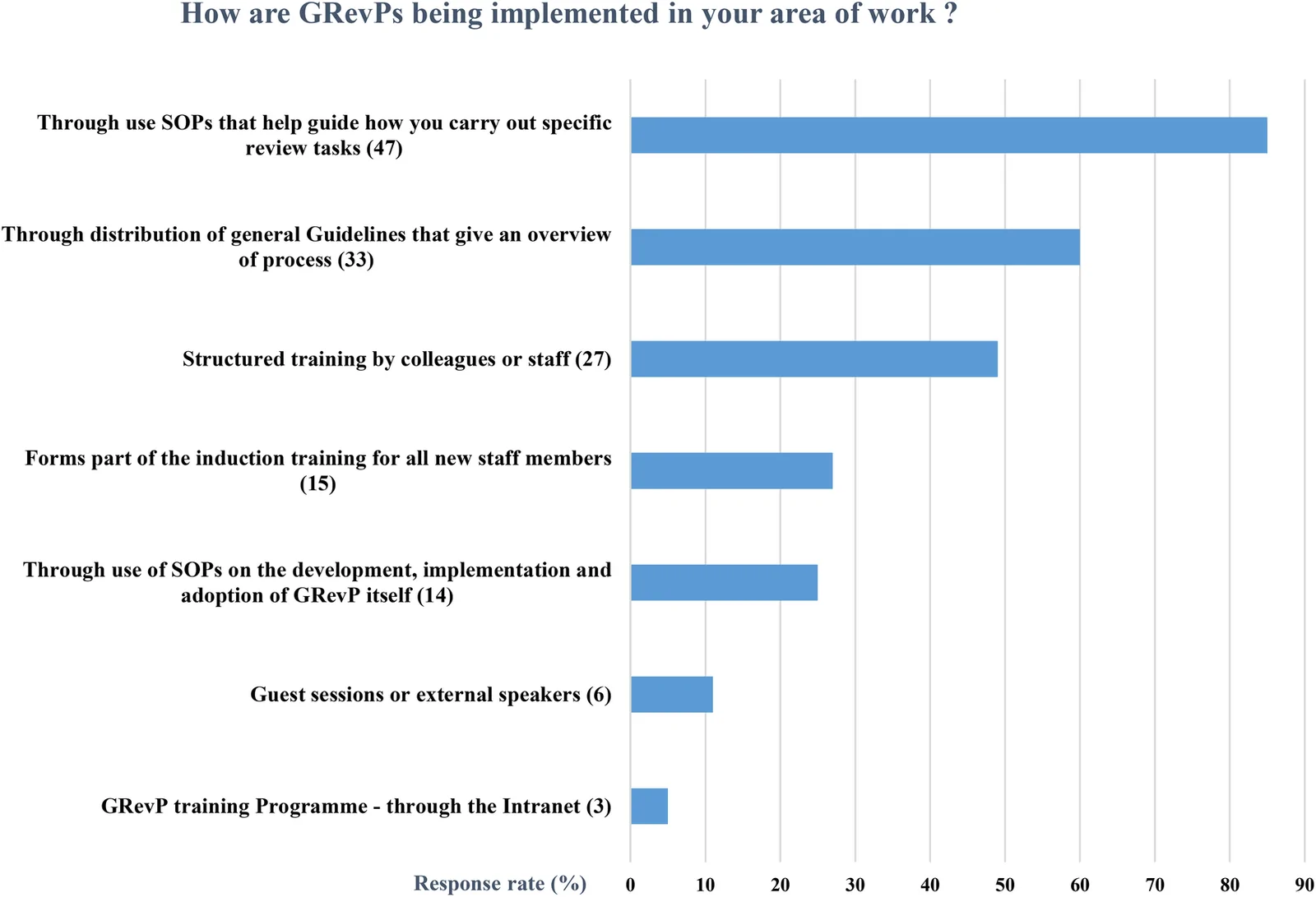
Mechanisms for implementing GRevPs within EDA. Note: Percentages are based on 55 respondents; numbers in parentheses indicate the number of respondents selecting each option. Multiple selections were allowed.

Formal quality audits (33%) and mentoring/supervision (30%) were the most frequently reported mechanisms used to monitor the consistent adoption and application of GRevPs. Training-related mechanisms were reported by 20% of assessors, whereas a smaller yet notable proportion (16%) indicated that no formal or informal mechanisms were in place.

Twenty-two assessors (40%) indicated that GRevP has been developed based on external best practices from other regulatory agencies, which are then adopted to the local context by EDA. Whereas 31% (*n* = 17) identified that the GRevPs have been developed by upper management review and quality teams, reflecting their view of the practices that should be adhered to. Further, 22%, (*n* = 12) indicated that GRevPs were based on collaborative best practices identified through the collective experience of EDA and review teams.

The majority of assessors (60%) reported being informed of new or updated GRevPs through self-service, with GRevPs guidance made available on SharePoint, the network drive, or the EDA website, and staff are responsible for identifying and using the latest versions. Sixteen assessors (29%) reported that updates were communicated during training to support consistent daily use. Notably**,** 7 (13%) assessors reported that it was unclear when GRevPs were developed or updated by the Agency, making it difficult to ensure the use of the most current guidance and standards.

### Part III—attitude

The majority (82%) indicated that the framework could be improved, while 13% reported being satisfied. Among those who selected “could be improved” or “not satisfied,” the most frequently cited reason (78%) was the need for additional training to better understand and integrate GRevPs into daily review activities. A further 18% indicated that the GRevPs system is still evolving and not yet fully developed. Only one respondent selected that the benefits of GRevPs are not yet apparent to the management or to themselves.

Figure 3 shows the perceived effectiveness of GRevPs in improving key aspects of the regulatory review process. Overall, the majority reported satisfactory improvements across multiple areas, including the quality of the review process, management of reviews, consistency, efficiency through standardisation, transparency, clarity of review procedures, and timeliness. These findings suggest that GRevPs are perceived to contribute positively to the EDA's review activities.

**Figure 3:**
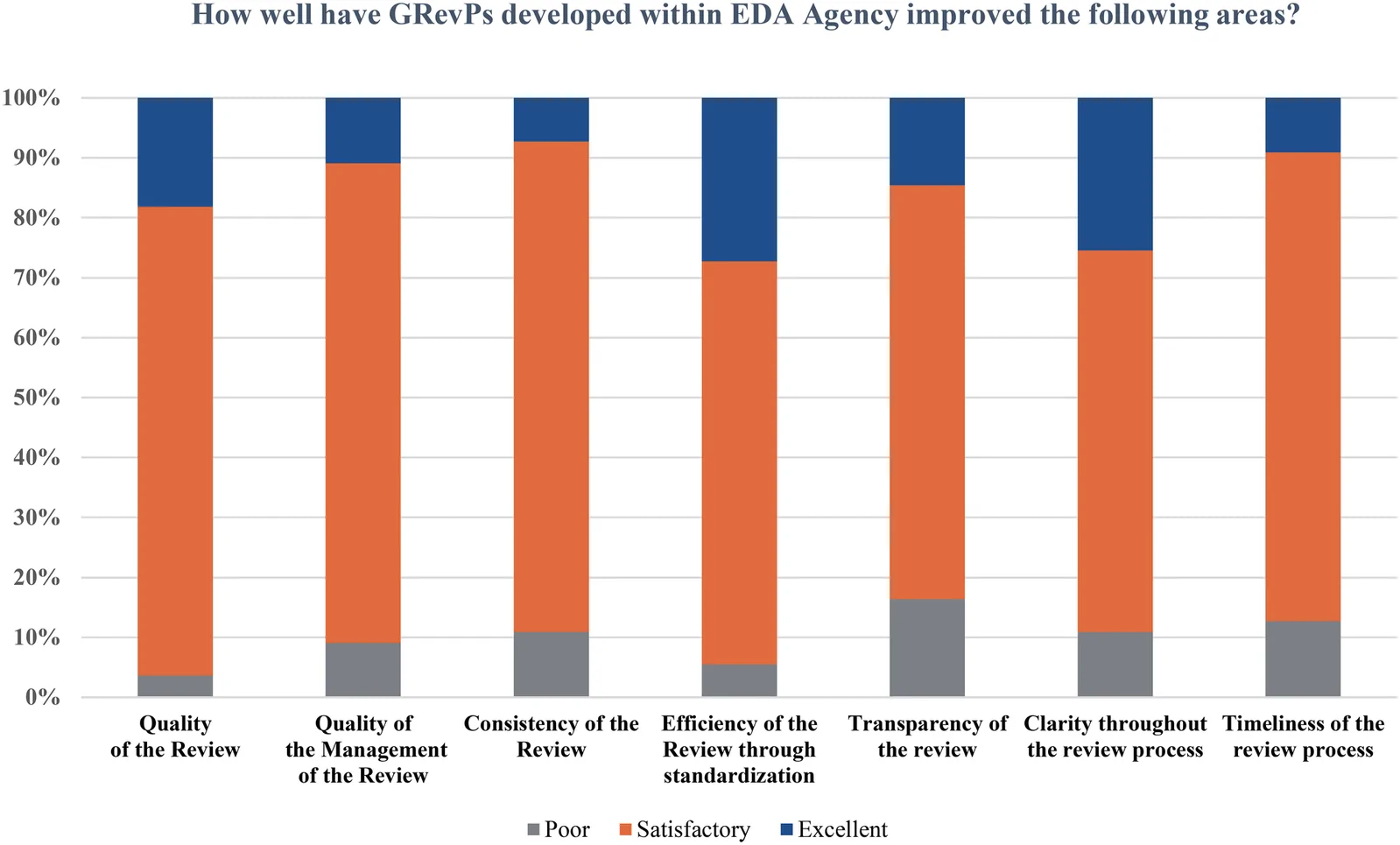
Rating of improvements associated with GRevPs within EDA.

The practical usefulness of GRevPs in supporting staff to perform review tasks effectively and achieve the EDA's goals is summarised in Table 3. For review principles and procedures, most EDA assessors reported receiving either some or strong guidance from existing GRevPs. Strong guidance was most frequently reported for review processes and methodologies (45%), science-based decision-making (44%), and decision-making communication (36%), indicating good support for core review functions. In contrast, risk control methodology showed the highest percentage of respondents reporting limited or no guidance (35%).

**Table 3:** Perceived helpfulness and level of guidance provided by GRevPs across review activities within EDA.

Review activity	No guidance *n* (%)	Some guidance *n* (%)	Strong guidance *n* (%)
Review procedures and principles			
Review processes & methodologies (decision-making)	0 (0%)	30 (54.6%)	25 (45.5%)
Multidisciplinary-based decision making	4 (7.3%)	33 (60.0%)	18 (32.7%)
Evidence-based decision	4 (7.3%)	27 (49.1%)	24 (43.6%)
Decision-making communication	5 (9.1%)	30 (546%)	20 (36.4%)
Risk control methodology	19 (34.6%)	28 (50.9%)	8 (14.6%)
Case management activities			
Internal Meetings	17 (30.9%)	28 (50.9%)	10 (18.2%)
Advisory meetings	5 (9.1%)	26 (47.3%)	24 (43.63%)
Communication with sponsors	7 (12.7%)	32 (58.2%)	16 (29.1%)
Conflict and dispute resolution	10 (18.2%)	39 (70.9%)	6 (10.9%)
Quality control	3 (5.5%)	33 (60.0%)	19 (34.6%)

Case management activities, which relate to the management and coordination of the review process, showed that most respondents received either some or strong guidance. Strong guidance was most frequently reported for advisory committee meetings (44%) and quality control (35%). However, “some guidance” remained the most common response across activities, with the highest rating for conflict and dispute resolution (71%). Internal meetings received the highest percentage (31%) of limited or no guidance.

### Part IV—gap analysis

The added gap analysis section showed good internal consistency, with Cronbach's alpha ranging from 0.85 to 0.91 across the six domains and activities. Content validity was also satisfactory, with an I-CVI and S-CVI of 1.00, as all three senior assessors rated every item as relevant.

To simplify results presentation and prioritise areas for improvement, gap scores were categorised into three levels using the calculated quartile thresholds. Values below Q1 (0.48) indicated good alignment between importance and implementation, values between Q1 (0.48) and Q3 (0.95) indicated moderate alignment requiring monitoring, and values at or above Q3 (0.95) indicated areas requiring prioritised improvement.

Across all assessed activities, implementation ratings were lower than importance ratings. The differences between perceived importance and implementation were statistically significant for all activities (*p* < 0.05), except for peer review, for which the difference was not statistically significant (*p* = 0.153). The largest gaps, in descending order, were continuous training, advance committee notification and proactive status updates for applicants, cross-team communication, documentation of meeting outcomes, internal electronic tracking, and consistent interpretation of regulatory requirements.

### Quality management activities

Core quality management tools, including standard operating procedures (SOPs), assessment templates and checklists, as well as peer review, showed good to moderate alignment between importance and implementation. It is worth noting that peer review had the smallest gap between importance and implementation across all 31 activities, with a score of 0.15 and was the only activity for which the difference between importance and implementation was not statistically significant (*p* = 0.153), followed by advisory committee engagement in the review conduct domain, with a gap score of 0.31. In contrast, larger gaps) (≥0.95) were observed for continuous training (1.62) and internal tracking systems (1.15), followed by feedback from applicants (0.96) indicating the need for improvement. The distribution of importance, implementation and gap scores across all activities is presented in Figure 4.

**Figure 4:**
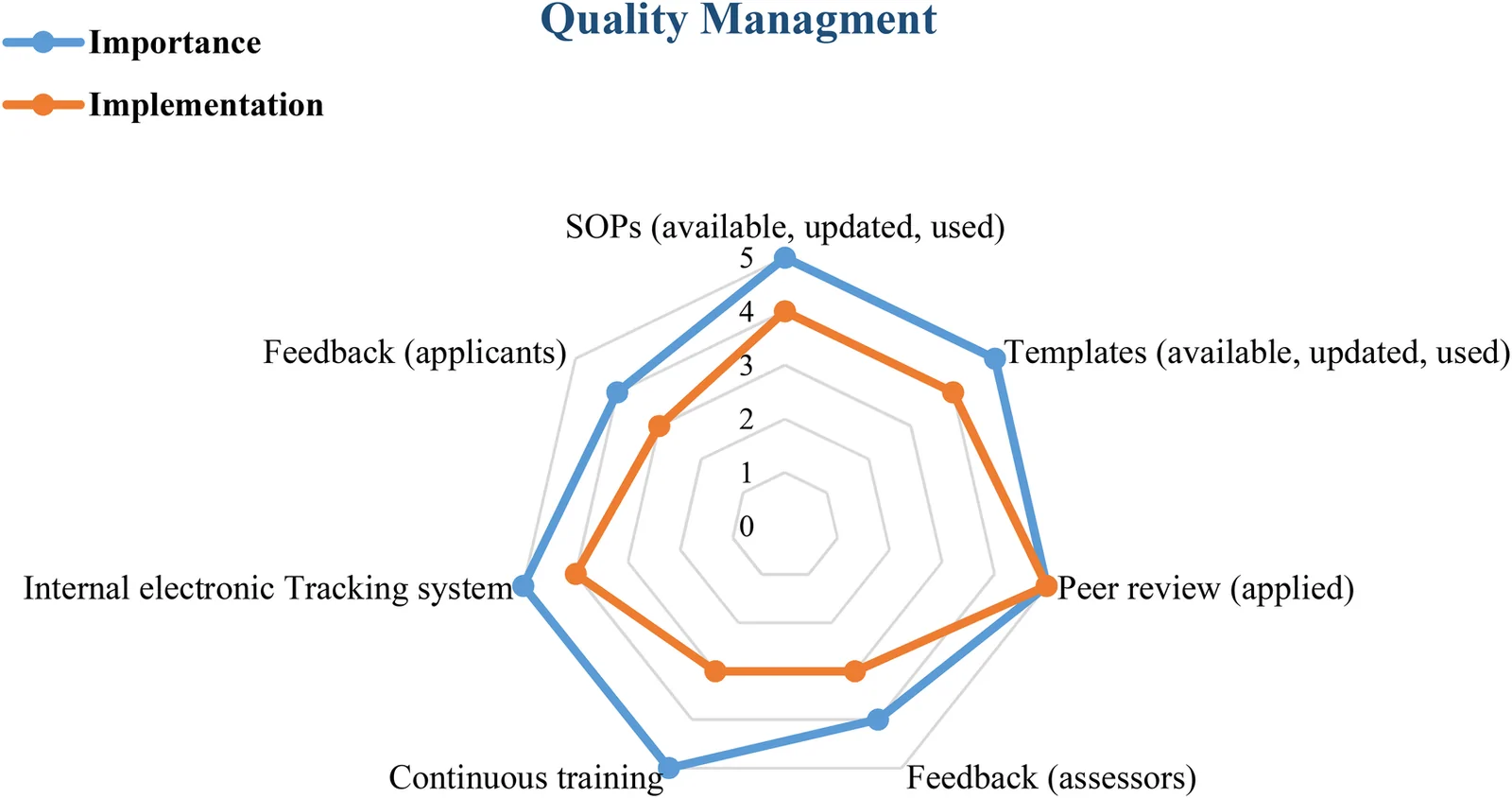
Gap analysis across quality management activities.

### Transparency and review conduct

Transparency-related activities were all rated as highly important and showed moderate alignment between importance and implementation, with gap scores ranging from 0.53 to 0.91, corresponding to published guidelines and the target timeline for review stages respectively.

Similarly, review conduct activities were rated highly important, with moderate gaps observed across most of them. The largest gap was identified for adherence to target timelines with a score of 0.94 (Figure 5).

**Figure 5:**
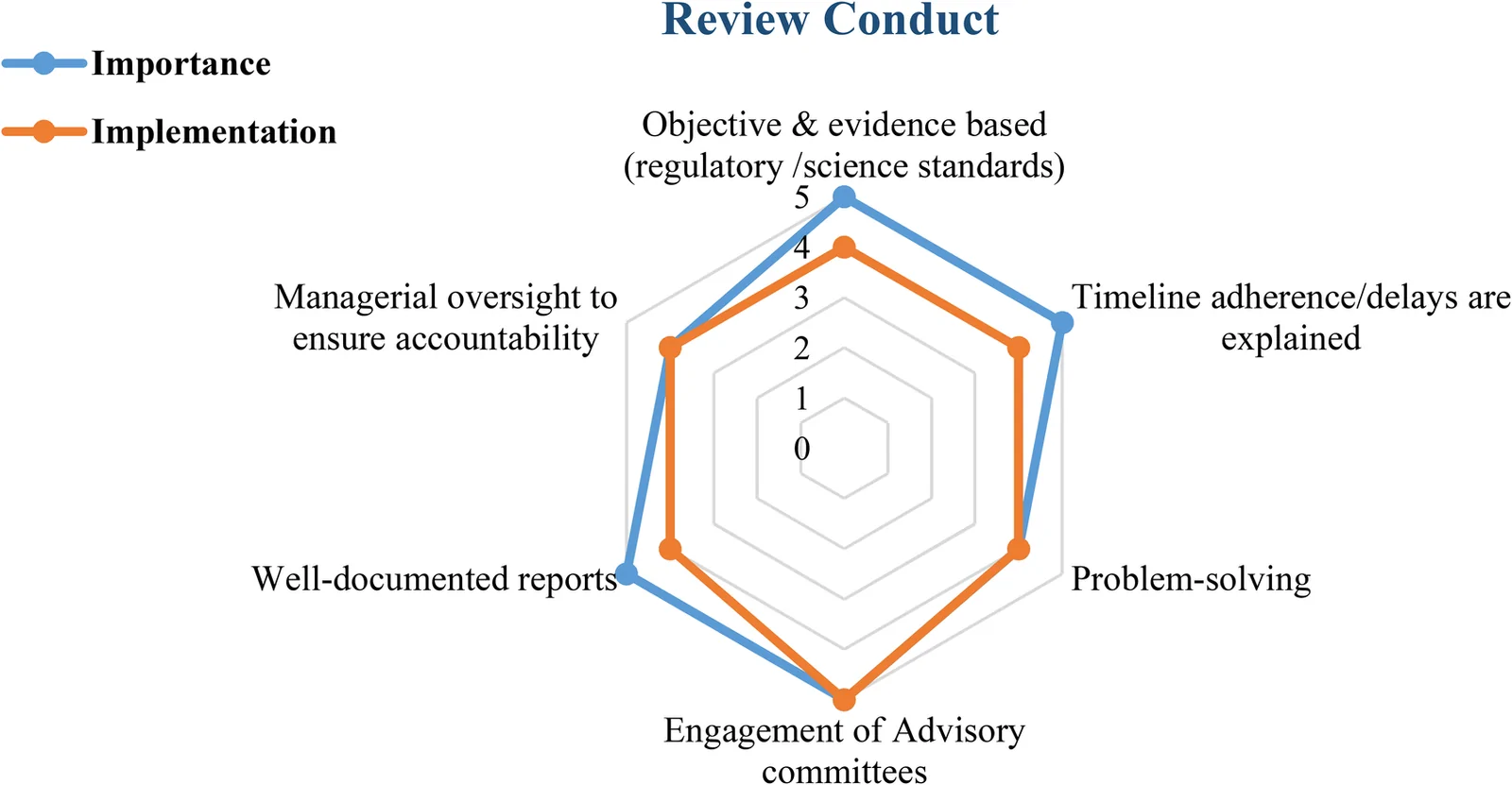
Gap analysis across review conduct activities.

### Consistency

The four consistency-related activities showed moderate to large gaps, ranging from 0.86 to 1.24. The largest gap was observed in communication and alignment between departments (1.24), followed by consistency in requirements interpretation (0.96). Moderate gaps were observed in maintaining consistency with previous regulatory precedents and in the consistency of review decisions across similar submissions.

### Communication

Communication activities related to formal pre-submission scientific advice, responsiveness to applicants through emails and phone calls and availability of meetings before and during submission showed good alignment, with gap scores below 0.48. In contrast, activities requiring proactive information sharing, including keeping applicants informed of application progress without needing to request updates, providing advance notice of committee meeting dates, and documenting meeting outcomes, showed larger gaps, ranging from 1.18 to 1.47 (Figure 6).

**Figure 6:**
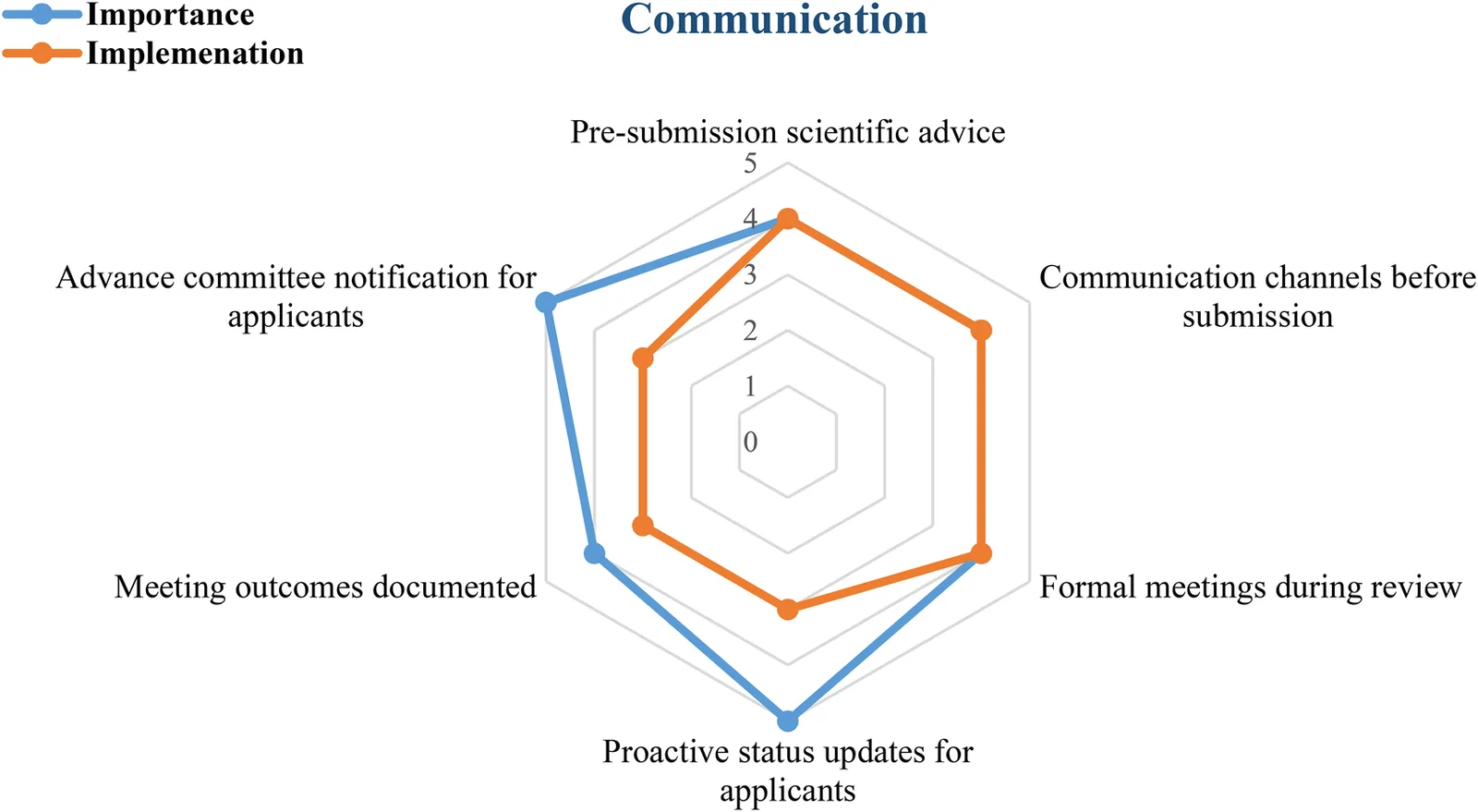
Gap analysis across communication activities.

### Stakeholder engagement

All stakeholder engagement activities were rated as important, with a mean gap score of 4. There was good alignment between importance and implementation, with the highest gap (0.6) observed for providing structured feedback to applicants on the quality of submitted dossiers.

### Supplementary importance–performance analysis (IPA)

The IPA matrix provided an additional assessment of areas requiring prioritised improvement across the assessed activities (Figure 7), with corresponding activity codes in Supplementary Table S1. Figure 7 presents the activities across the four quadrants of the IPA matrix. The upper-left quadrant represents activities with high importance but lower implementation (Concentrate here), while the upper-right quadrant represents activities with high importance and high implementation (Keep up the good work). The lower-left quadrant includes activities with lower importance and lower implementation (Low priority), whereas the lower-right quadrant includes activities with lower importance but higher implementation (Possible overkill). Seven activities were positioned in the quadrant, representing high importance and low implementation. Five of these were also identified as priority areas in the arithmetic gap analysis: continuous training, advance committee notification for applicants, proactive status updates for applicants, effective communication and alignment of requirements across teams and departments, and the internal electronic tracking system. The remaining two activities were consistency of decisions across similar submissions and problem-solving in review conduct.

**Figure 7:**
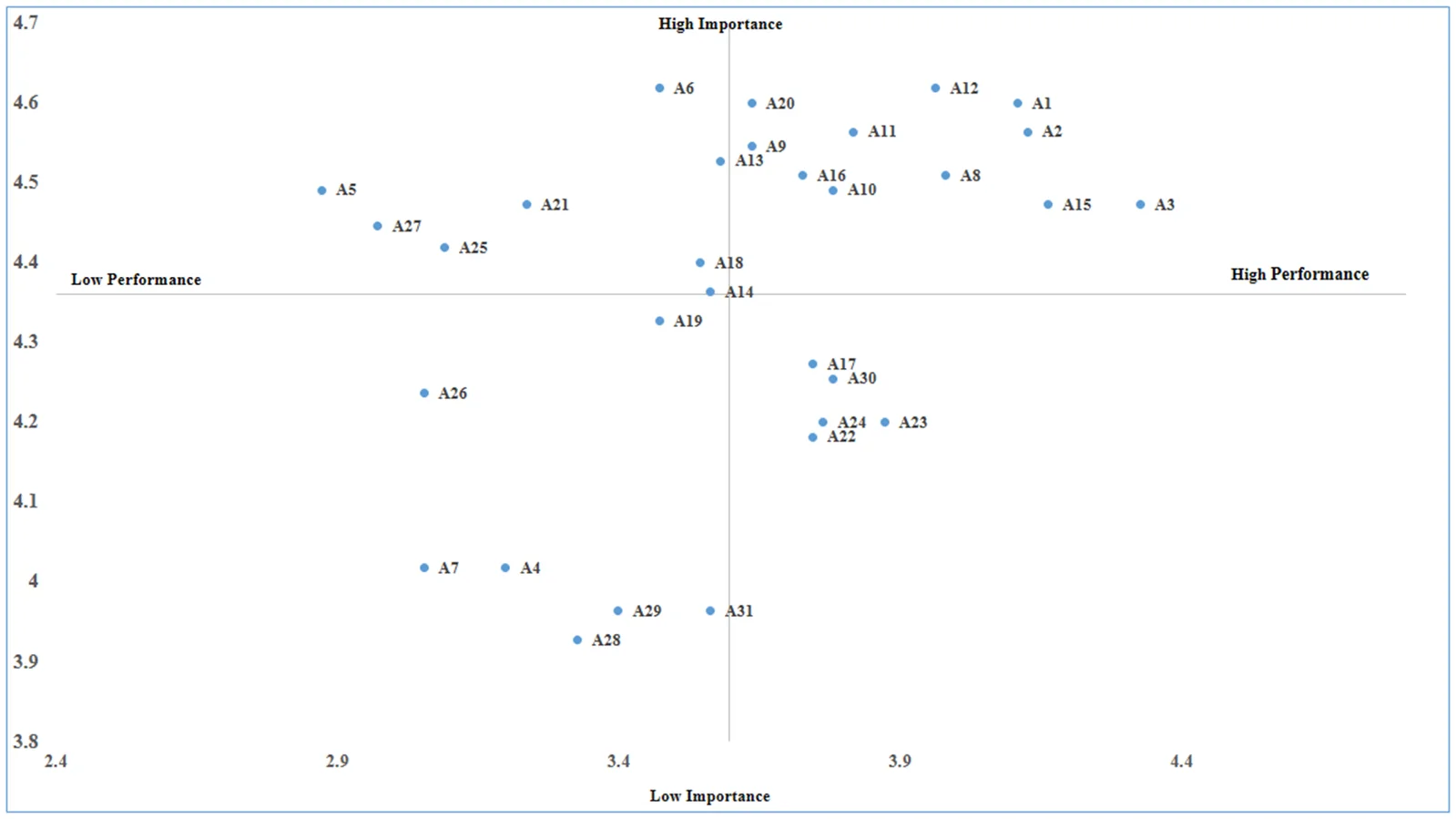
Importance–performance analysis (IPA) matrix.

### Part V-perceived barriers to GRevPs implementations

Time pressure was the most frequently selected barrier, with 80% indicating that high workload and tight deadlines limit the ability to apply GRevPs thoroughly. In addition, 67% of respondents perceived some GRevP-related activities, such as completing checklists and documenting meeting minutes, as time-consuming administrative work rather than part of the scientific review process. Around 60% also reported limitations in IT systems, including document management and tracking tools, as well as insufficient staff and resources. A smaller proportion of respondents noted limited feedback on the effectiveness of GRevPs (34%) and found some guidelines and tools unclear or complex (38%), which may affect their use in daily review work.

### Part VI-qualitative insights

#### Theme 1. Awareness and understanding of GRevPs

Comments highlighted differences in awareness and understanding of GRevPs across departments, particularly outside the registration functions. Assessor 3 commented:

“Some departments away from registration don't fully know and understand the concepts of GRevPs, while others excel in it.”

Some comments also indicated that understanding of GRevP principles and practices remained inconsistent across departments and among assessors. Activities such as sharing committee meeting dates and assessment criteria were still perceived by some colleagues as internal matters rather than as part of transparency practices and applicants’ rights.

#### Theme 2. Training and capacity-building

##### Training access

Training was one of the most frequently reported challenges in the open-ended responses. Assessors noted that training was not always continuous or equally accessible across staff and highlighted the need for regular refresher activities to reinforce the consistent application of GRevPs in routine practice.

##### Training content

Comments also showed they need for training that combines technical and quality-related aspects of regulatory review, with a stronger focus on practical training and case studies rather than theoretical sessions. Assessor 13 commented:

“What problem-solving skills shall I acquire and implement while reviewing CTD module one?”

#### Theme 3. Workload and resource constraints

Assessors’ comments highlighted high workload, staff shortages, and resource constraints as challenges affecting GRevPs implementation, including adherence to review timelines, communication with applicants, and documentation of review steps. Assessor 42 commented:

“High workload and low resources lead to poor outcomes.”

#### Theme 4: internal communication and coordination

Comments highlighted challenges in communication, coordination, information sharing across departments, as well as inconsistent understanding and interpretation of regulatory requirements

#### Theme 5: digitalisation and process improvement

Manual processes were described as consuming time that could otherwise be spent on review activities**.** Digitalisation was considered important for improving review efficiency. Assessor 6 commented:

“Consider digital transformation seriously to improve review process and timeline adherence”

## Discussion

This study assessed the extent to which Good Review Practices (GRevPs) are embedded within the Egyptian Drug Authority (EDA), based on the perceptions and experiences of Marketing Authorisation (MA) assessors. The findings provide insights into assessors’ awareness and understanding of GRevPs, as well as the measures, activities, and tools in place to build and sustain quality in the review process. Overall, the results establish a baseline for EDA's GRevPs implementation, identifying strengths and gaps as the agency advances toward regulatory maturity level 4 and WHO-Listed Authority (WLA) status.

The study showed that within MA departments, comparable proportions of respondents reported full and partial adoption of GRevPs (44% and 53%, respectively). However, adoption across the wider authority was mostly perceived as partial. This indicates that GRevPs are not yet consistently applied across all departments, assessors, and tasks, and that broader organisational integration remains an ongoing process. Similar findings have been reported in other African regulatory authorities, despite differing in organisational structure, staffing capacity, resources, and maturity levels. In Zambia, 42% of assessors reported full adoption of GRevPs (15), while 70% of respondents in the Ghana FDA study reported full adoption (14).

Perceived GRevPs adoption at both the MA department and the wider EDA varied significantly by assessors’ role level and years of regulatory experience, with full adoption more frequently reported among more senior and experienced assessors. These findings should be interpreted cautiously given the small subgroup sizes and the inability to adequately adjust for potential confounding factors.

EDA assessors demonstrated a good understanding of the purpose and value of GRevPs, and of their contribution to improving review efficiency and consistency, reducing duplication, and minimising errors. Similar findings were reported in the Ghana FDA study, where efficiency, consistency, and error reduction were identified as the main drivers for implementing quality measures (14). It has been acknowledged that a good understanding of GRevPs influences assessors’ practices and attitudes and promotes their effective implementation (14).

Furthermore, GRevPs in EDA are developed based on practices adopted from other regulatory authorities and international organisations, reflecting the authority’s commitment to aligning with global standards while adapting them to the local context, consistent with WHO guidance (7).

The reported good alignment between perceived importance and implementation within the quality management and review conduct domains suggests that the core procedural elements of GRevPs are well integrated into EDA's routine review practices. The use of standard operating procedures, assessment templates, multidisciplinary scientific assessment, and peer review reflects the adoption of the key building blocks of Good Review Practices (9). Similarly, transparency appears to be embedded within the EDA review process. Publicly available guidelines, defined review timelines and criteria, and the publication of assessment reports with decision rationales enable applicants and other stakeholders to better understand regulatory expectations and the basis for regulatory decisions, thereby supporting confidence in those decisions (7). Notably, EDA is among the few African regulatory authorities currently publishing public assessment reports and the rationale for final decisions, whereas several previous African studies noted limited implementation of this practice (15).

Importantly, the study identified that several GRevPs activities require further strengthening. These include continuous training, review consistency, adherence to target timelines, consistent recording and tracking of review activities, proactive communication with applicants, cross-team coordination, and mechanisms for translating collected feedback into improvement actions. All of these activities were recognised by assessors as important, indicating that awareness of GRevPs is well established. However, assessors perceived that their consistent implementation was more difficult under barriers of high workload, time pressure, and limited staffing capacity. This perception is consistent with findings from Zambia, where inadequate human resource capacity contributed to difficulties in adhering to target timelines and, consequently, delays in patients’ timely access to medicines (15).

Several of the identified gaps appear closely interlinked, with improvements in one activity likely to strengthen performance in others. Continuous training is essential for consistent scientific assessments, effective application of GRevPs, and keeping pace with evolving regulatory practices (2). Furthermore, consistent recording and tracking of review activities support routine monitoring of the review process, contributing to adherence to the target timelines (28). Similarly, feedback mechanisms contribute to continuous quality improvement only when the information collected is analysed and translated into improvement actions (10).

An important area identified for further improvement is communication. Effective communication between departments supports alignment of technical requirements (7), while proactive communication with applicants should extend beyond responding to applications and inquiries. Providing applicants with information on committee meeting schedules, review progress, and expected timelines are reported to improve the predictability and efficiency of the review process (28).

Documentation-related activities, including checklist completion and meeting documentation, were perceived by some assessors as administrative requirements rather than integrated components of the scientific review process, despite their role in documenting review activities, decisions, and follow-up actions.

The gaps identified in this study are consistent with findings from Ghana FDA (14), the Zambia Medicines Regulatory Authority (ZAMRA) (15), the Economic Community of West African States Medicines Regulatory Harmonization initiative (ECOWAS-MRH) (29), and the East African Community Medicines Regulatory Harmonization initiative (EAC-MRH) (28), where ongoing challenges were similarly reported in communication practices, such as advisory committee meeting dates communication, feedback mechanisms, adherence to timelines, transparency activities, and human resource capacity. Regulatory authorities progressing toward higher maturity levels are increasingly expected to implement proactive applicant communication, performance monitoring, and effective electronic tracking systems to support transparency and oversight throughout the review process (7, 10).

Within this context, the findings indicate that EDA has established many of the foundational elements required for implementation of GRevPs, supported by documented procedures, review tools, and organisational quality structures. The findings also demonstrate a good level of understanding of quality review practices within the MA functions. Together, these findings align with the foundational “say what you do” phase described in the WHO GRevPs guidance, where review processes and procedures are formally defined and documented (7). However, greater attention is still required in the subsequent phases: “do what you say,” “prove it,” and “improve it”, to support consistent implementation, continuous monitoring, and improvement activities across regulatory functions. This reflects the continuing challenge of moving from establishing GRevP procedures to fully embedding them in routine regulatory practice.

### Recommendations

The following recommendations are informed by the gap analysis, with priority given to activities with gap scores at or above 0.95. The EDA should provide structured, mandatory GRevP training, with greater emphasis on practice-oriented, case-based training and regular refresher courses. Training should be consistently delivered across departments and updated to reflect evolving regulatory practices. Provide ongoing scientific and technical training to maintain assessors’ competencies in their respective areas of review. To strengthen proactive communication, implement an external electronic tracking system with automated notifications so applicants can monitor application progress and receive timely updates.

Internally, regular departmental meetings should be organised to improve communication and alignment across teams, supported by digital platforms and shared workspaces for information sharing between departments. Assessors within the same team should regularly discuss review cases and relevant previous decisions to promote a common understanding and consistent interpretation of regulatory requirements. Meeting outcomes should clearly document the issues discussed, decisions made, and agreed actions. For meetings with applicants, this record should be shared to provide clarity on the discussion and agreed next steps.

Although not part of the formal gap analysis, assessors’ comments also highlighted the need to strengthen workforce capacity, staff retention, and resource allocation to sustain GRevPs and maintain review quality and institutional knowledge. Finally, monitoring mechanisms should be strengthened to support the effective implementation of GRevPs and improvement actions. This should include consistent recording of key review milestones in the existing internal tracking system, together with regular audits and review of assessment reports to monitor adherence to established guidelines, SOPs, regulatory requirements, and target timelines. Feedback from assessors and applicants should also be systematically collected and analysed, with findings translated into actions and followed up to evaluate their implementation and effectiveness.

### Study limitations

Several limitations should be acknowledged. Although the original GRevP questionnaire is a validated tool, the construct validity of the 31 newly added gap analysis activities could not be assessed because the sample size did not meet the recommended 5:1 respondent-to-item ratio (30) However, content validity was assessed by three experts (S-CVI = 1.00), and internal consistency was good (Cronbach's *α* = 0.85–0.91). The cross-sectional design captured assessors’ perceptions at a single point in time and could not assess changes in GRevP implementation over time. In addition, self-reported perceptions may be subject to response bias and may not fully reflect actual regulatory practices. Therefore, as this is a cross-sectional perception-based study, the results cannot establish causal relationships between GRevP implementation and regulatory performance. Participation was voluntary, which may have introduced selection bias and limited the generalisability of the findings. The small numbers within some subgroups also limited multivariable analysis and the ability to account for potential confounding factors. Finally, comparisons with other regulatory authorities in the discussion section should be interpreted cautiously, as differences in organisational structure, staffing capacity, and regulatory systems may influence GRevP implementation.

### Future research

Despite these limitations, the findings establish a foundation for future research with larger, more diverse samples across the agency. Future studies could benefit from including a wider range of departments (e.g., vigilance, inspection, laboratory), collecting additional demographic characteristics such as postgraduate qualifications and participation in international training programmes, and expanding the survey across the entire agency. This broader approach would enable more comprehensive multivariable analyses and enhance the generalizability of findings.

## Conclusion

This study assessed the implementation and embedding of GRevPs within the EDA from the perspective of marketing authorisation assessors and provided baseline insights into assessors’ knowledge, perceptions, and implementation practices related to quality review activities. The findings demonstrate that EDA has established many of the foundational elements required for implementation of GRevPs, while also identifying operational areas requiring further improvement, particularly in consistent implementation, monitoring, communication, and continuous improvement activities. These findings may support ongoing regulatory strengthening efforts as EDA progresses toward maturity level 4 and WHO-Listed Authority aspirations.

## Data Availability

The original contributions presented in the study are included in the article/Supplementary Material, further inquiries can be directed to the corresponding author.
