# Supplementary material for: An evaluation of perceptions and implementation of good review practices in the medicine review process at the Egyptian drug authority
Source: Front Med (Lausanne). 2026 Oct 8;13:1920714. doi: 10.3389/fmed.2026.1920714 (PMC13687002; doi:10.3389/fmed.2026.1920714)
Supplement: Supplementary file 1 [file Table1.docx]

| **Code** | **Activity** | **Domain** | **Mean Importance** | **Mean performance** | **Gap** | **P value** | **IPA Matrix Quadrant** |
| --- | --- | --- | --- | --- | --- | --- | --- |
| A5 | **Continuous training** | Quality management | 4.491 | 2.873 | 1.618 | <0.05 | I - Concentrate Here |
| A27 | **Advance committee notification for applicants** | communication | 4.445 | 2.973 | 1.473 | <0.05 | I - Concentrate Here |
| A25 | **Proactive status updates for applicants** | communication | 4.418 | 3.091 | 1.327 | <0.05 | I - Concentrate Here |
| A21 | **Effective communication and alignment of requirments across teams and departments** | Consistency | 4.473 | 3.236 | 1.236 | <0.05 | I - Concentrate Here |
| A6 | **Internal electronic Tracking system** | Quality management | 4.618 | 3.473 | 1.145 | <0.05 | I - Concentrate Here |
| A26 | **Meeting outcomes shared** | communication | 4.236 | 3.055 | 1.182 | <0.05 | III - Low Priority |
| A20 | **Consistent interpretation of requirments within the team** | Consistency | 4.600 | 3.636 | 0.964 | <0.05 | II - Keep Up the Good Work |
| A13 | **Timeline adherence/delays are explained** | Review Conduct Items | 4.527 | 3.582 | 0.945 | <0.05 | II - Keep Up the Good Work |
| A9 | **Target timelines published** | Transparency | 4.545 | 3.636 | 0.909 | <0.05 | II - Keep Up the Good Work |
| A7 | **Feedback (applicants)** | Quality management | 4.018 | 3.055 | 0.964 | <0.05 | III - Low Priority |
| A18 | **Consistency of review decisions across similar submissions.** | Consistency | 4.400 | 3.545 | 0.855 | <0.05 | I - Concentrate Here |
| A19 | **Past cases and regulatory precedents are considered** | Consistency | 4.327 | 3.473 | 0.855 | <0.05 | III - Low Priority |
| A16 | **Well-documented reports** | Review Conduct Items | 4.509 | 3.727 | 0.782 | <0.05 | II - Keep Up the Good Work |
| A14 | **Problem-solving** | Review Conduct Items | 4.364 | 3.564 | 0.800 | <0.05 | I - Concentrate Here |
| A11 | **Assessment reports published** | Transparency | 4.564 | 3.818 | 0.745 | <0.05 | II - Keep Up the Good Work |
| A4 | **Feedback (assessors)** | Quality management | 4.018 | 3.200 | 0.818 | <0.05 | III - Low Priority |
| A10 | **Decision criteria published** | Review Conduct Items | 4.491 | 3.782 | 0.709 | <0.05 | II - Keep Up the Good Work |
| A12 | **Objective & evidence based (regulatory /science standards)** | Review Conduct Items | 4.618 | 3.964 | 0.655 | <0.05 | II - Keep Up the Good Work |
| A8 | **Published guideline/requirements/procedures** | Transparency | 4.509 | 3.982 | 0.527 | <0.05 | II - Keep Up the Good Work |
| A28 | **Structured feedback on dossier quality** | Stakeholder engagement | 3.927 | 3.327 | 0.600 | <0.05 | III - Low Priority |
| A1 | **SOPs (available, updated, used)** | Quality management | 4.600 | 4.109 | 0.491 | <0.05 | II - Keep Up the Good Work |
| A17 | **Managerial oversight to ensure accountability** | Review Conduct Items | 4.273 | 3.745 | 0.527 | <0.05 | IV - Possible Overkill |
| A29 | **Formal appeal mechanisms** | Stakeholder engagement | 3.964 | 3.400 | 0.564 | <0.05 | III - Low Priority |
| A30 | **Applicant engagement in guidance development** | Stakeholder engagement | 4.255 | 3.782 | 0.473 | <0.05 | IV - Possible Overkill |
| A2 | **Templates (available, updated, used)** | Quality management | 4.564 | 4.127 | 0.436 | <0.05 | II - Keep Up the Good Work |
| A24 | **Formal meetings during review** | Commuincation | 4.200 | 3.764 | 0.436 | <0.05 | IV - Possible Overkill |
| A22 | **Pre-submission scientific advice** | Commuincation | 4.182 | 3.745 | 0.436 | <0.05 | IV - Possible Overkill |
| A31 | **Public initiatives/patient feedback** | Stakeholder engagement | 3.964 | 3.564 | 0.400 | <0.05 | III - Low Priority |
| A15 | **Engagement of Advisory committees** | Review Conduct Items | 4.473 | 4.164 | 0.309 | <0.05 | II - Keep Up the Good Work |
| A23 | **Communication channels before submission** | Communication | 4.200 | 3.873 | 0.327 | <0.05 | IV - Possible Overkill |
| A3 | **Peer review (applied)** | Quality management | 4.473 | 4.327 | 0.145 | 0.1528 | No stat significant |
| **Overall Mean** | |  | 4.359 | 3.5757 |  |  |  |

**Table S1: Distribution of assessed activities by gap score and Importance–Performance Analysis (IPA) quadrant. Activities are presented in descending order of gap score. The overall mean importance and implementation scores are shown in the final row.**
